# Ciliary vortex flows and oxygen dynamics in the coral boundary layer

**DOI:** 10.1038/s41598-020-64420-7

**Published:** 2020-05-05

**Authors:** Cesar O. Pacherres, Soeren Ahmerkamp, Gertraud M. Schmidt-Grieb, Moritz Holtappels, Claudio Richter

**Affiliations:** 1 Alfred Wegener Institute, Helmholtz Centre for Polar and Marine Research, Bremerhaven, Germany; 2 University of Bremen, Bremen, Germany; 3 Max Planck Institute for Marine Microbiology, Bremen, Germany; 4 Marum, Bremen, Germany

**Keywords:** Ecophysiology, Fluid dynamics, Biological physics, Biophysics

## Abstract

The exchange of metabolites between environment and coral tissue depends on the flux across the diffusive boundary layer (DBL) surrounding the tissue. Cilia covering the coral tissue have been shown to create vortices that enhance mixing in the DBL in stagnant water. To study the role of cilia under simulated ambient currents, we designed a new light-sheet microscopy based flow chamber setup. Microparticle velocimetry was combined with high-resolution oxygen profiling in the coral *Porites lutea* under varying current and light conditions with natural and arrested cilia beating. Cilia-generated vortices in the lower DBL mitigated extreme oxygen concentrations close to the tissue surface. Under light and arrested cilia, oxygen surplus at the tissue surface increased to 350 µM above ambient, in contrast to 25 µM under ciliary beating. Oxygen shortage in darkness decreased from 120 µM (cilia arrested) to 86 µM (cilia active) below ambient. Ciliary redistribution of oxygen had no effect on the photosynthetic efficiency of the photosymbionts and overall oxygen flux across the DBL indicating that oxygen production and consumption was not affected. We found that corals actively change their environment and suggest that ciliary flows serve predominantly as a homeostatic control mechanism which may play a crucial role in coral stress response and resilience.

## Introduction

It is commonly perceived that sessile aquatic organisms are subjected to the physicochemical conditions of their environment, and that their capacity to cope and adapt determines their survival^1^. It is less known, however, that sedentary animals may also shape their environment physically and chemically^2,3^. In these cases, active responses through adaptation or modifications of the environment may entail large energetic costs, particularly in small organisms operating at small Reynolds numbers, where energy reserves are low. In the case of coral colonies, which individuals have limited or no motility, modifying the characteristics of their immediate surrounding, i.e. the Diffusive Boundary Layer (DBL), might be crucial for their survival^4^.

The DBL is the thin layer of water adjacent to all submerged surfaces where molecular diffusion is the dominant mechanism of transport for dissolved substances. The flow in the DBL is laminar and parallel to the boundary surface, so that vertical advective fluxes approach zero and diffusive fluxes dominate. The DBL is generally between 0.2–1 mm thick^5^ and varies depending on the flow speed of the ambient water^6–8^. Its upper boundary is defined by the distance where the eddy diffusion coefficient, *K*, which governs the turbulent free-flow region of the water column, approaches the molecular diffusion coefficient, *D*^9^. Therefore, and according to classic boundary layer theory, assuming that there is no production or consumption of the compound within the DBL itself, the concentration gradient across the DBL should be linear^10,11^.

In most coral colonies, the individual polyps are small (cm to mm) and surrounded by a DBL, the thickness of which depends on ambient water flow and colony morphology^12,13^. Any exchange or transport of dissolved substances between organism and surrounding water column has to happen via diffusion through this DBL, a process that in turn will depend on the molecules’ diffusivity and concentration gradients^14^. For tropical corals living in symbiosis with unicellular dinoflagellate algae (Zooxanthellae), oxygen is of particular importance. During the day, the photosynthetic algae produce a surplus of oxygen, as a function of light intensity^15,16^, zooxanthellae density^17^, ambient flow speed^18,19^, and dissolved inorganic carbon^20^. This excess of oxygen accumulates in the tissue and diffuses across the DBL into the water column aloft^21^. During the night, oxygen is consumed reversing the direction of the diffusive flux from the water column towards the tissue^22^. The characteristics of the DBL, therefore, have a strong influence on coral physiology and its ability to respond to ambient conditions.

The water flow in the DBL is laminar and parallel to the surface of the solid^23^. However, in corals the cilia covering the tissue have the ability to enhance the mixing in this layer by generating vortices^4^. These cilia are present in most Scleractinian corals^24^ and considered to be important as feeding and cleaning mechanisms, where they transport particles towards and/or away from the oral openings^25–27^. So far, the mixing-effect of ciliary vortices have been described by carrying out PIV and microsensor measurements under stagnant flow conditions and constant light, and extending the findings to natural flow conditions by modelling^4^. Here, we extend this study to measure the changes in oxygen across the DBL at different flow speeds, light regimes and under active and arrested cilia activity (the latter achieved using sodium orthovanadate^4^ – see M&M), using the reef-building coral *Porites lutea* as a model organism. Particle Image Velocimetry (PIV) and Particle Tracking Velocimetry (PTV) were used to map the microscale flow regime at the coral surface and to identify vortex locations when present. Microsensor probes were used to obtain oxygen profiles across the DBL and vortices. Chlorophyll fluorescence quenching was used to measure the effect of ciliary flows on coral health.

## Material and Methods

### Coral fragments

Colonies of the massive coral *Porites lutea* reared at the aquaria facilities of the Alfred Wegener Institute (AWI) under conditions mimicking those found at the depth of their origin (15 m) were used as fragment source^28,29^. They were kept in artificial seawater (salinity 32.6 ± 0.26) (Dupla Marin Premium Reef Salt Natural Balance) at 25.2 ± 0.07 °C on a 12-h light-dark cycle. Light intensities fluctuated between 75 and 80 μmol quanta m^−2^ s^−1^ (LI-COR LI-192, USA) and pH was kept at 7.9 ± 0.09 (YSI, USA). Food was provided in the form of freshly hatched *Artemia* nauplii every second day. Before the start of the experiments, small fragments (1.5 cm long, 1 cm wide) bearing 60 to 80 polyps were cut out from 4 of the source colonies and allowed to heal for at least two weeks in the same culturing tanks their original colonies were kept. Survival of the fragments was >90%.

### Experimental set up

**Figure 1:**
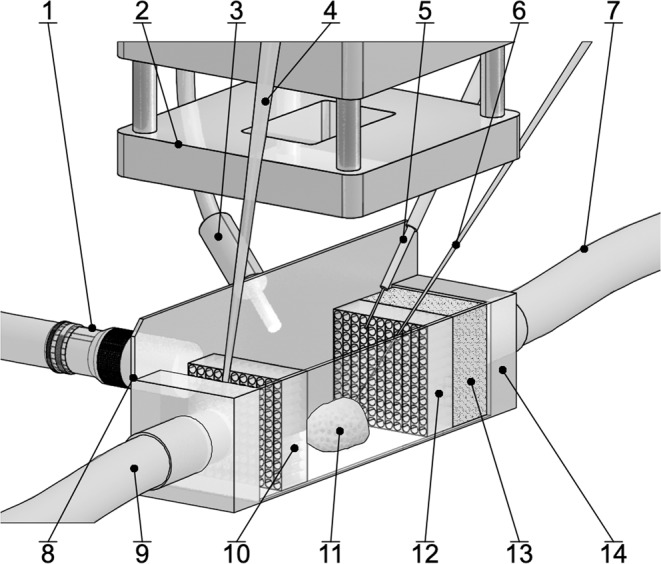
Schematic illustration of the light-sheet-microscopy based flow chamber setup. The coral fragment (11) was placed at the center of the PDMS chamber (14) with microscopy glass slides as walls (8). Homogeneous water flow was achieved by placing a sponge (13) and a diffuser (12) at the inlet (7) and a diffuser (10) at the outlet (9). A fiber optic lamp (3) was used as light source for all experiments under light conditions. The LED lens (2) projected a 1 mm thick light sheet on top of the coral colony for the PIV and PTV measurements carried out by a camera pointing sideways and equipped with a long distance microscope lens (1). An electrochemical sensor (6) was used for the oxygen profiles. Temperature (4) and optical oxygen (5) sensors were used to monitor water characteristics inside the chamber. Figure not drawn to scale.

A polydimethylsiloxane (PDMS) flow-through chamber (2 cm long × 1.5 cm wide × 1.5 cm high) was built over microscope glass slides to allow a side view of the coral colony while leaving an open space on top to allow access for the microsensors (Fig. 1). The chamber was connected to a water pump (REGLO Z, ISMATEC, Germany) by 0.5 mm ∅ silicon hoses. Homogeneous water flow inside the chamber was achieved by placing a sponge on the water inlet followed by a diffuser with a series of 1 mm holes and by placing a second diffuser on the outlet of the chamber.

The coral fragment was placed in the chamber filled with Filtered Artificial Sea Water (FASW) (0.2 pore size) from the coral´s culturing tank. It was allowed to acclimate to the chamber conditions under a low flow regime (water replacement in the chamber every 2 minutes) for at least an hour under dark conditions before experiments started.

To arrest cilia activity, we used the fully reversible ATPase inhibitor sodium orthovanadate (Na_3_VO_4_; Sigma Aldrich). Prior to the experiment, the coral fragment was kept for an hour on FASW containing Na_3_VO_4_ at a concentration of 0.1 mM^30^. Arrest of cilia activity was verified visually under a stereo microscope before the fragment was transferred into the experimental flow-through chamber supplied with Na_3_VO_4_–treated FASW for the duration of the experiment.

Oxygen and temperature of the water inside the chamber were monitored every 30 seconds using optical sensors (microoptodes, PyroScience, Germany) positioned at the inlet and outlet of the chamber, respectively (Fig. 1). For all experiments, temperature was kept constant at 25  ± 0.5 °C and oxygen at 100% saturation.

### Particle velocimetry

To resolve the flow field around the coral fragment, Particle Image Velocimetry (PIV) and Particle Tracking Velocimetry (PTV) were used. Water inside the flow through system was first seeded with 5 μm fluorescent particles (Exc: 468 nm, Emi: 530 nm) (Applied Microspheres, The Netherlands). Particle density was adjusted to approx. 50 × 10^6^ particles L^−1^, to allow a good map of the flow field without inducing mucus production, a natural response in corals to suspended sediments^25^. Illumination was achieved using a LED Pulsing System PIV V2 (ILA_5150, Germany) connected to LED light sheet optics^31^. The light sheet was 1 mm thick and intensity reached 5500 μmol quanta m^−2^ s^−1^ (LI-COR LI-192, USA) at a wavelength of around 468 nm. Images were captured using a ILA.PIV.μEYE camera (ILA_5150, Germany) recording pictures at 25 frames per second (fps) with an exposure time of 26 ms. A long distance microscope lens (Optem FUSION, Germany) with a bandpass filter of 532 nm was used for all experiments. To avoid photoinhibition of the zooxanthellae from the intense illumination, light exposure was <5 s for each experiment.

Video frames were processed using PIV software (PIVview2C, PIVTech GmbH). To obtain velocity fields, the image sample size was set to 64 × 64 px with a 50% overlap and a multigrid sampling window of 128 × 128 px. The velocity map was smoothed using a 3 × 3 median filter. PIV output was then post-processed using custom-built Matlab (MathWorks, R2018b) algorithms to obtain the averaged velocity components. Particle trajectories (PTV) were obtained by superimposing consecutive images and the corresponding velocities were calculated by implementing a particle tracking algorithm (Matlab).

### Oxygen profiles

Dissolved oxygen profiles in the DBL were carried out using OX10 oxygen microelectrodes (Unisense, Denmark) connected to an OXY-meter amplifier (Unisense, Denmark). Values were recorded using the SensorTrace-PRO software (Unisense, Denmark). Each day, microelectrodes were 2-point calibrated in oxygen-free (bubbling pre-filtered seawater with nitrogen gas for 10 min) and air-saturated FASW of known salinity and temperature^21^. The tip of the sensor was carefully placed at the surface of the coral. A micromanipulator (Unisense, Denmark) was programmed to move the sensor up in 10 µm steps. The range of the vertical profile was 1000 µm. At each step, dissolved oxygen was measured three times with a sampling interval of 2 s. Oxygen profiles were processed using R Studio Version 1.1.4.

### Determination of oxygen flux and DBL thickness

The oxygen flux across the DBL was calculated from the measured oxygen profiles using Fick’s first law of diffusion:

$$J=-D\frac{\partial C}{\partial z}$$

where D is the molecular diffusion coefficient of oxygen at a specific temperature and salinity, in our case: 2.13 × 10^−9^ m^−2^ s^−1^ ^32^ and $\frac{\partial C}{\partial z}$ is the vertical oxygen concentration gradient, calculated by linear regression, where *C* is the concentration and *z* the vertical distance.

Commonly, the upper boundary of the DBL is determined by the intercept between the linear concentration gradient and the constant part of the O_2_ profile, representing the bulk concentration in the free-stream water aloft^5^. The lower boundary in this study was the coral surface, but only for the case of no cilia activity. Shapiro *et al*.^4^ showed that ciliary vortices modified the oxygen transport in the lower boundary layer, creating curved or S-shaped profiles that indicate an effective mass transport coefficient significantly larger than the molecular diffusion coefficient. For flux calculations we therefore used the linear part of the profiles above the vortices, a layer we refer to as upper DBL, where particle tracks indicate a laminar, plane parallel flow (see results below) and in which a predominantly diffusive transport can be assumed. We further assume that oxygen is neither produced/consumed in the vortex layer nor in the upper DBL so that the oxygen flux across the two adjacent layers is equal and ultimately represents the flux between coral and ambient water.

### Photosynthetic efficiency

The kinetics of chlorophyll fluorescence is commonly used to assert the efficiency of the photosynthetic apparatus, which is susceptible to environmental stress^33,34^ by measuring the maximum quantum yield (MQY) of the Photosystem II complex (PSII) (*Fv/Fm*). We evaluated the response of the coral´s PSII to the experimental manipulation of flow and light under active and arrested ciliary motion by Pulse Amplitude Modulation (PAM) chlorophyll *a* fluorometry^35^, using a diving PAM fluorometer (Walz, Germany). For this purpose, three coral fragments were subjected to all experimental conditions and the maximum quantum yield of PSII measured for each treatment combination, allowing a 15 min adaptation of the coral fragment to the light/dark conditions. PAM data was processed using R Studio.

### Experimental design

To identify the relative importance of light, flow speed and cilia activity on the oxygen profiles of *Porites*, their effects were tested in combination. All experiments were performed in random order and with different coral fragments. Different flow rates from the pump were chosen, resulting in flow velocities of 300, 425, 750 and 1300 µm s^−1^ at 2 mm above the coral tissue (Fig. S1). Such velocities in the laminar boundary layer correspond to flow velocities of 1-2 cm s^−1^ in a turbulent boundary layer above estimated from Van Driest^36^ which compare with the flow velocities measured in the immediate vicinity of coral colonies^12^.

For all experiments under light, a fiber optic lamp (Schott 1500, USA) was used as light source to maintain constant illumination of 165 μmol quanta m^−2^ s^−1^. For the experiments under dark conditions (<1 μmol quanta m^−2^ s^−1^), all lights were turned off 15 minutes prior to the start of the oxygen profiles. Oxygen profiles were recorded 15 min after the PIV measurement.

## Results

### Flow field and DBL

**Figure 2:**
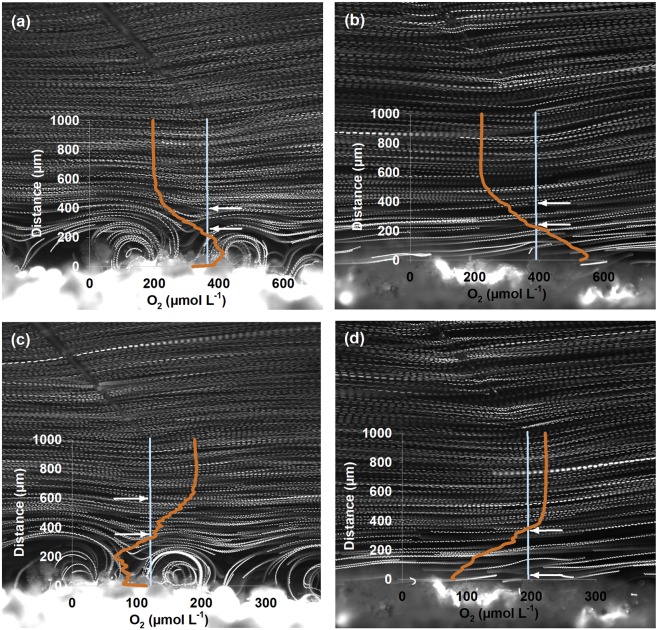
Pathline image and oxygen profiles of the coral *Porites lutea* under active (**a**,**c**) and arrested (**b**,**d**) cilia activity as well as under light (**a**,**b**) and dark (**c**,**d**) conditions. Pathlines indicate particle trajectories. Orange lines indicate oxygen concentration along the light blue line measured with a microsensor (also seen in the images). Flow speed of water in this case was 1300 µm s^−1^ measured at 2 mm from the coral surface (see text for more details). Arrows indicate the part of the diffusive boundary layer (DBL) that was considered for oxygen flux calculations.

The particle trajectories of the PIV measurements revealed laminar flow conditions in the boundary layer of the coral with water flowing parallel to the tissue surface. These conditions prevailed throughout the entire boundary layer whenever cilia activity was suppressed (Fig. 2b,d). When cilia were active, vortices near the tissue were detected, under light exposure and in the dark (Fig. 2a,c). The vortex diameters, as approximated from the particle trajectories, were 200 µm under high flow velocities (1300 µm s^−1^, Fig. 2a,c) and 500 µm under low flow velocities (300 µm s^−1^, Fig. S2a,c). The upper boundary of the DBL varied inversely with flow. At low flow velocities, the DBL extended up to 1000 µm above the coral surface, while at high velocities it was reduced to 500 µm.

### Oxygen gradients and fluxes

**Figure 3:**
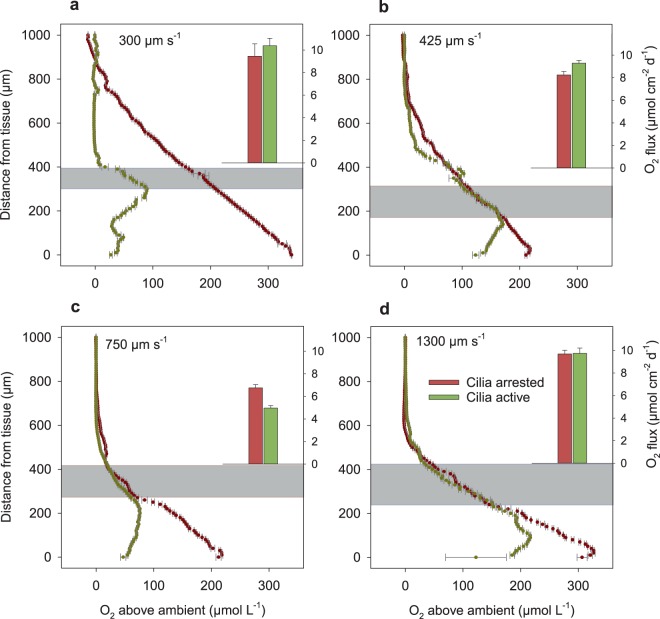
Oxygen concentrations along a perpendicular 1000 µm transect from the tissue of* Porites lutea* to the water column under light exposure, at flow speeds of (**a**) 300, (**b**) 425, (**c**) 750 and (**d**) 1300 µm s^−1^, and for arrested and active cilia conditions. Inserted bar plots show the flux of oxygen as calculated from oxygen gradients in the upper DBL (grey layer). Error bars represent ± standard error of the linear regression.

With arrested cilia and under light, photosynthetic oxygen production caused very high oxygen concentrations near the coral surface (300 µM above saturation), which decreased linearly from the surface to the upper boundary of the DBL (Fig. 2b). When cilia were actively beating, the induced vortices were mixing the lower boundary layer thereby reducing and partly reversing the oxygen gradients (Fig. 2a). Vortex mixing caused a decrease of oxygen concentrations at the coral surface by about 110 µM. Above the vortex layer, the plane parallel particle trajectories and the linear oxygen gradients indicated a diffusive transport. The oxygen gradient in the upper DBL was comparable to the gradient measured under arrested cilia (Fig. 3d), suggesting that the flux across the DBL did not change by cilia activity.

For the other flow velocities (Fig. 3a–c), the oxygen gradients in the upper DBL were similar with and without cilia activity, but in all cases the total oxygen content in the DBL was strongly reduced, thus decreasing the extreme oxygen concentrations near the coral surface.

The oxygen extremes were less pronounced in the dark than in light. With arrested cilia and under dark conditions (Fig. 2d), we found a linear oxygen decrease by 140 µM between the upper boundary of the DBL and the tissue surface. Under cilia activity (Fig. 2c), vortices were again mixing the lower boundary layer. The generation of vortices was associated with a reduced oxygen shortage near the coral surface (only 75 µM below ambient concentrations). For the lower flow velocities (Figs. S2b,d and S3), the arrested cilia condition caused a linear decrease in oxygen concentration towards the tissue surface. The oxygen respiration in the dark caused much less deviation from ambient concentrations compared to oxygen production under light exposure. During cilia activity, the vortices caused a redistribution of the oxygen in the vortex layer, which however was not always related to a significant relaxation of oxygen condition towards ambient concentrations.

The oxygen concentration gradient above the vortices was used to calculate the oxygen flux across the upper DBL. The undisturbed upper DBL started where the particle trajectories were quasi-parallel to the tissue surface and extended until the oxygen concentration reached that one of the bulk water (see Fig. 2a,c). If possible, the oxygen gradients measured under arrested and active cilia were taken from the same length interval to calculate the flux across the upper DBL (grey layers in Fig. 3). Under light exposure, the calculated fluxes ranged between 5 and 10 µmol cm^−2^ d^−1^ and appeared to show no relation to flow velocities (Fig. 3, inserts). The fluxes were comparable between arrested and active cilia conditions with no significant difference at low and high flow velocities (300 and 1300 µm s^−1^) and only small differences for the intermediate velocities (425 and 750 µm s^−1^).

When oxygen was consumed by the holobiont in the dark, the oxygen profiles show less overlap between arrested and active cilia conditions (Fig. S3). Again, only those concentration gradients were considered for flux calculations that were situated in the plane parallel flow, either above the vortices or closer to the tissue surface where a linear gradient was established during arrested cilia conditions. The resulting fluxes ranged between −3.7 and −7.3 µmol cm^−2^ d^−1^. Except for the low flow velocity, the fluxes were significantly different between arrested and active cilia conditions. However, no trend was found as only in two cases the flux was enhanced by the cilia activity (Fig. S3).

### Oxygen concentrations in the vortex layer

**Figure 4:**
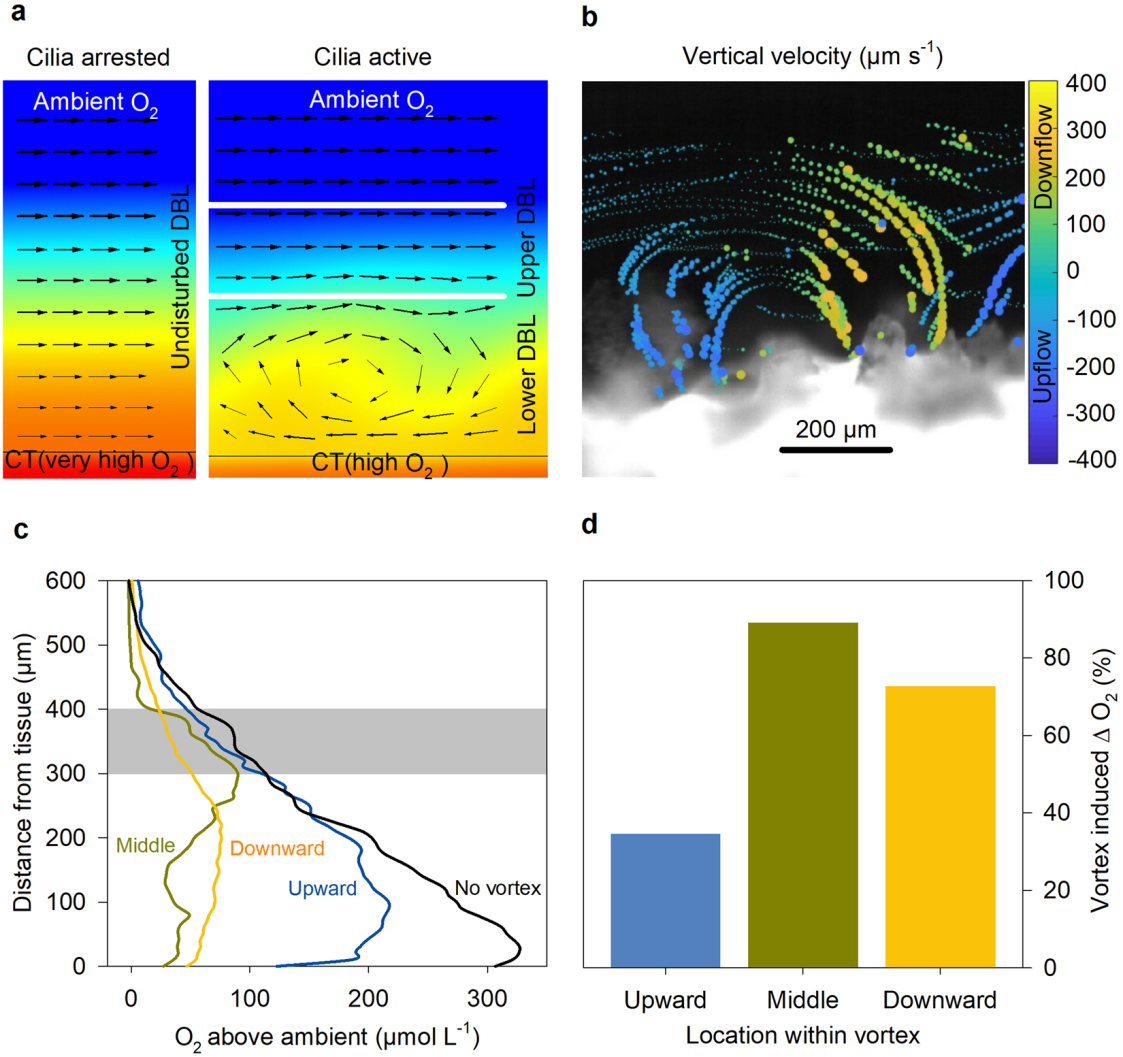
(**a**) Schematic drawing of the enhanced transport mechanism within the vortex layer. Colors denote oxygen concentrations, arrows indicate the flow field. The oxygen gradient close to the tissue is undisturbed when cilia are arrested (left). A revolving vortex (right) destabilizes the gradient by advecting high concentrations upwards and low concentration downwards. Just above the vortex, the exchange is again dominated by diffusion. We define this layer as upper DBL. The enhanced transport by the vortex causes decreased oxygen concentrations close to the coral tissue (CT). (**b**) Detailed vertical velocity component of a vortex extracted from the PIV results under 1300 µm s^−1^ flow speed, positive values indicate movement towards the coral while negative values indicate water moving away from the coral. (**c**) Compilation of the oxygen profiles plotted by their location within the vortex: blue -upward section (Fig. 3d - Flow speed 1300 µm s^−1^); green - middle section (Fig. 3a - Flow speed 300 µm s^−1^) and yellow - downward section (Fig. 3c - Flow speed 750 µm s^−1^), black line represents the profile under arrested cilia activity (Fig. 3d - Flow speed 1300 µm s^−1^). Gray area shows the slope similarities of the profiles regardless of flow speed and cilia activity. Vortex extension must be taken under consideration when interpreting the graph. (**d**) Vortex induced delta O_2_ under light conditions for the different locations within the vortex. The reduction of oxygen concentration in the first 50 µm of the DBL under active cilia is shown in percent of the above-ambient oxygen concentrations measured under arrested cilia.

In the vortex layer close to the coral tissue, the shape of the oxygen profile was dependent on the position of the microsensor relative to the 2-dimensional vortex geometry. For profile interpretation, the vortex mixing is briefly described (Fig. 4a). The cilia-generated vortices can be seen as stationary features that enhance the exchange between the coral tissue and the upper DBL where the ambient water is flowing plane-parallel. When the corals’ photosymbionts are producing oxygen, the lower part of the vortex is exposed to high oxygen concentrations at the coral tissue so that oxygen diffuses into the lower vortex. As the vortex revolves, elevated oxygen concentrations are transported to the upper part of the vortex, which is exposed to the relatively low oxygen concentration in the upper DBL, so that oxygen diffuses out of the upper vortex. On the downturn, the vortex brings again relatively low oxygen to the coral tissue. This revolving distribution mechanism is reflected in the shape of the oxygen profiles (Fig. 4b,c). The largest dissimilarity to the arrested state was seen for profiles that intersected the downward flow and close to the middle of the vortex, where relatively low oxygen concentrations are transported downwards. The least change was found in the upward flow of the vortex where relatively high concentrations are transported upwards. The flow speeds in the boundary layer did not seem to affect the overall pattern.

While the effect of cilia activity on the oxygen fluxes across the upper DBL is minor, vortices strongly affect the oxygen concentrations close to the tissue. The differences in oxygen concentrations were strongest during light conditions, where a decrease of up to several hundred micromoles per liter was caused by cilia activity (Fig. 3). In order to quantify the oxygen mitigation close to the tissue, the oxygen concentrations in the lower 50 µm, i.e. below the vortex center and above 10 µm from the tissue (to avoid any boundary artifacts), were averaged for arrested and active cilia conditions. Subsequently the deviations from ambient oxygen concentrations were compared (averaging over the lower 100 µm shows similar results). Under light conditions, cilia activity reduced the above-ambient concentration at the tissue by 34-89% (Fig. 4d). The average reduction over the three vortex positions (middle position, downward and upward flow position) was 60%.

### Photosynthetic efficiency

**Figure 5:**
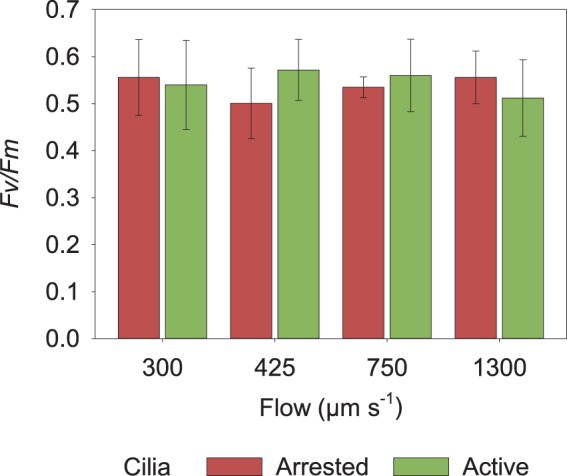
Maximum quantum yield (MQY) of photosystem II (*Fv/Fm*) of zooxanthellae within *Porites lutea* in relation to the flow speed of the water (µm s^−1^) and cilia activity (active and arrested) after 15 min light adaptation (165 μmol quanta m^−2^ s^−1^). Error bars represent ± s.d.

The photosynthetic efficiency was measured for three coral fragments to assess the environmental stress due to the various experimental conditions. *Fv/Fm* values were similar across all experiments (Fig. 5). No differences were detected due to light regimes, flow speeds or cilia activity, tested neither as single factors nor in interactions (ANOVA, Table S1). Moreover, there was no effect of the ciliary inhibitor on the photosynthetic efficiency of the zooxanthellae as can be seen on the similar MQY values when compared active and arrested cilia activity under light (Fig. 5) (For the MQY values in the dark, see Supplementary Fig. S4).

## Discussion

Corals have the ability to create a microhabitat around them with characteristics that can be considerably different from those of the water column^37^. The flow and oxygen measurements revealed a clear effect of cilia-induced vortex flow on oxygen concentration in the boundary layer of the coral *Porites lutea* under natural flow and light conditions. The vortices enhanced the mixing of oxygen in the lower boundary layer regardless of the ambient flow speed and therefore significantly changed the oxygen concentrations close to the coral tissue compared to the diffusion-controlled transport without cilia activity. Vortex mixing is a complex transport process in which advection and diffusion are intertwined. If vortices are assumed stable over time, the water parcels revolve permanently without an effective advection out of the vortex. However, as the water parcels in a vortex revolve, they are brought to layers of different oxygen concentrations so that steep concentration gradients enhance the diffusive exchange. The transport across the vortex layer is thus a mixture of diffusion and advection which, on average, results in an increased effective mass transport coefficient larger than the molecular diffusion coefficient. Above the vortex layer, the laminar flow was oriented plane parallel so that diffusive transport was again dominating the vertical flux. The vortex layer was thus topped by an upper diffusive boundary layer in which oxygen gradients could be measured and fluxes determined. Since we assume no O_2_ production or consumption in the vortex layer and in the upper DBL, the flux across the two layers is equal and ultimately represents the flux between coral and ambient water. In the light exposure experiments, the gradients and thus the fluxes in the (upper) DBL were similar with and without cilia activity (Fig. 3) suggesting for both cases a constant flux and thus a constant O_2_ production in the coral tissue. The enhanced mass transport coefficient in the vortex layer did not result in enhanced fluxes, indicating that photosynthetic oxygen production of the endosymbionts was neither down- nor up-regulated upon vortex mixing but remained constant, seen also in the PAM results, and probably depended only on light intensity and symbiont density^15,17^.

In our study, the main benefit of the generated vortices is the reduction of elevated oxygen concentrations at the immediate surface of the coral. The vortex mixing had thus a different outcome in our measurements compared to the Shapiro-Fernandez model^4^. The reason is that the Shapiro-Fernandez model assumed constant oxygen concentration at the coral tissue, which results in increased oxygen fluxes upon cilia activity, whereas we observed constant fluxes which results in reduced concentrations. Nevertheless, the relative increase of the mass transport coefficient due to cilia activity is comparable in both studies. In our study, the effect of vortices can roughly be estimated from the reduction in oxygen concentrations close to the tissue. Upon vortex mixing, the oxygen concentrations at the coral surface decreased on average to 40% of the above-ambient concentration without vortex mixing. Applying Fick’s law for the entire boundary layer (i.e. vortex layer and upper BL) and assuming a constant flux (*J*), a reduction of concentrations by a factor of 0.4 suggests a reciprocal enhancement of the effective mass transport coefficient (*D*) by a factor of 2.5 (=1/0.4). This compares well with the 2-3 fold flux enhancement that was modeled for a small-polyped plate under similar flow conditions^4^. Under the observed constant O_2_ production, the role of vortex mixing is predominantly the mitigation of excessive oxygen concentrations at the coral tissue. A change of oxygen concentrations at the tissue surface has several physiological consequences. Oxygen is the essential element for all aerobic organisms. However, as in the case of corals, excess of oxygen can also be detrimental to living tissues^38,39^. The higher affinity of Rubisco Form II to oxygen over CO_2_ will cause photorespiration, which has a high metabolic cost^40^. On the other hand, the production and accumulation of oxygen radicals after photosynthesis have proved to cause cellular damage and a subsequent bleaching reaction in corals^41–43^. Vortex formation caused the mitigation of the high oxygen values seen under arrested cilia activity, improving the conditions at the tissue surface by promoting the efflux of oxygen out of the tissue and reducing its detrimental consequences inside the coral. We suggest that cilia-induced flows change the chemical environment of corals as part of a homeostatic control mechanism. This mechanism may be of particular importance for buffering the activity of the photosynthetic endosymbionts whose oxygen production may not be downregulated by the host organism itself.

The cilia-mediated mitigation should be also effective for other substrates and products that are exchanged with the ambient water. Assuming the same ratio between the fluxes of oxygen and protons as found by Chan, *et al*.^44^ (12 µmol O_2_ cm^−2^ d^−1^ and −7.28 10^−10^ mol H^+^ cm^−2^ d^−1^), we calculated that for an ambient pH of 7.9, the expected pH at the tissue surface in our experiments would have been 8.53 pH units without vortex mixing. Cilia induced vortices would have effectively reduced the pH to 8.06 units. Photosynthesis, respiration and calcification influence proton concentration at the surface of the coral^45^ with higher pH values during light regimes. Cilia activity will cause the mitigation of extreme proton depletion at surface levels, enhancing their flux towards the tissue, which in turn might favor coral´s growth rates.

The results were less conclusive when oxygen was consumed by the holobiont in the dark. Although the vortex flows clearly affect oxygen gradients in the lower DBL, the calculated fluxes were neither similar between active and arrested cilia conditions nor was there a clear trend suggesting an enhancement of the flux. The mitigation of oxygen depletion by active cilia was less obvious and found only in 2 out of 4 flow velocities. We suggest two possible reasons for the deviating results under dark conditions. First, different from the oxygen production which was controlled mostly by light intensity and endosymbiont density, the consumption of the holobiont may be diffusion limited within the tissue, depending on the tissue thickness and the oxygen demand therein. Enhanced oxygen concentrations under active cilia conditions would result in enhanced fluxes. Second is the potential heterogeneity within the investigated coral tissue and between different coral fragments. Different coral fragments were used for each flow field and oxygen measurement, and the tissue itself, its thickness and oxygen demand may be less homogenous compared to the endosymbiont distribution^46^. The oxygen demand of the holobiont may also depend on temporal aspects such as day-night rhythm or the time period since the last feeding. Nevertheless, it is likely that the enhanced vortex mixing is also beneficial in the dark during oxygen consumption. Corals living in reef flats and lagoons can experience periods of minimum water flow^47^ which can lead to frequent and prolonged periods of hypoxia^21^ causing bleaching and death of the coral colony^48^. Cilia beating at night might therefore help to alleviate detrimental low oxygen concentrations, which together with other mechanisms such as polyp expansion, triggered by low light^49^ will promote the exchange of oxygen with the water column^22^.

For the interpretation of the results we assume that the experimental treatments did not affect the photosynthetic efficiency as the *Fv/Fm* of the coral colonies during the experiments was in the range of the response of healthy corals in the field (0.5 −0.9)^50^. There was not a significant change of the photosynthetic efficiency of PSII as a result of the exposure of the coral fragments to neither sodium orthovanadate (used to inactivate cilia movements), nor to light. Corals under high light conditions tend to present lower yield values reflecting the stress the PSII is being exposed to^51^ however in our case, light intensity was not as strong as to produce a detectable stress signal in the PSII efficiency, which, at the same time, excludes photoinhibition due to oxygen radical production^41^.

Further research is needed to study the response of cilia activity to different boundary conditions under elevated heat, increased pH, or high particle concentrations. Ideally, the 1-dimensional oxygen profiling should be supported by 2-D or 3-D visualization of oxygen concentrations such as shown by Koren *et al*.^52^ who captured the oxygen dynamic on the tissue surface of a larger coral fragment. Moreover, the possible involuntary arrestment of cilia beating by different stressors requires as well in-depth understanding, since elemental accumulation or depletion at tissue level might have detrimental consequences to coral physiology. Understanding the physico-chemical conditions of the coral’s DBL and the way the organism interacts with that layer is essential if we want to elucidate its response to a changing environment. The proven ability of corals to alter their DBL and, by doing so, interfere with diffusion processes consequently shaping their environment, might be the missing key when trying to understand processes such as selective bleaching and recovery in the face of warming and acidifying oceans.

## Supplementary information

Supplementary information.
