# Supplementary material for: Ciliary vortex flows and oxygen dynamics in the coral boundary layer
Source: Sci Rep. 2020 May 5;10:7541. doi: 10.1038/s41598-020-64420-7 (PMC7200650; doi:10.1038/s41598-020-64420-7)
Supplement: Supplementary file 1 — Supplementary information. [file 41598_2020_64420_MOESM1_ESM.pdf]

SUPPLEMENTARY INFORMATION

Ciliary vortex flows and oxygen dynamics in the coral boundary layer

Authors:

Cesar O. Pacherres[1,2,*], Soeren Ahmerkamp[3,4], Gertraud M. Schmidt-Grieb[1], Moritz Holtappels[1,4,a], Claudio Richter[1,2,a]

Affiliations:

1 Alfred Wegener Institute, Helmholtz Centre for Polar and Marine Research, Bremerhaven, Germany

2 University of Bremen, Germany

3 Max Planck Institute for Marine Microbiology, Bremen, Germany

4 Marum, Bremen, Germany

a These authors share senior authorship

* Corresponding author

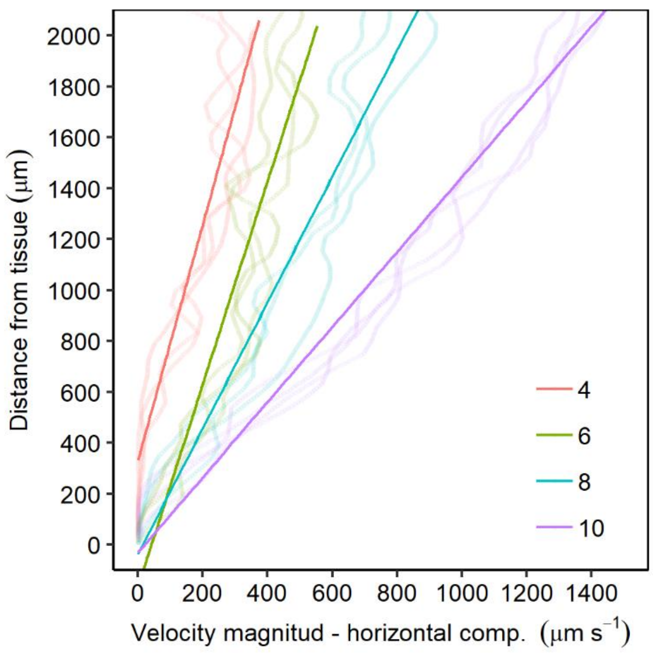

Fig. S1 Horizontal flow speeds (μm s^{-1}) parallel to the coral surface according to the four pump rates chosen for the experiments: 4, 6, 8, 10 (mL min^{-1}). Solid line represents the linear fit of the different flow speeds profiles. Background curves correspond to the individual velocity profiles extracted from the PIV of the different experiments under arrested cilia activity.

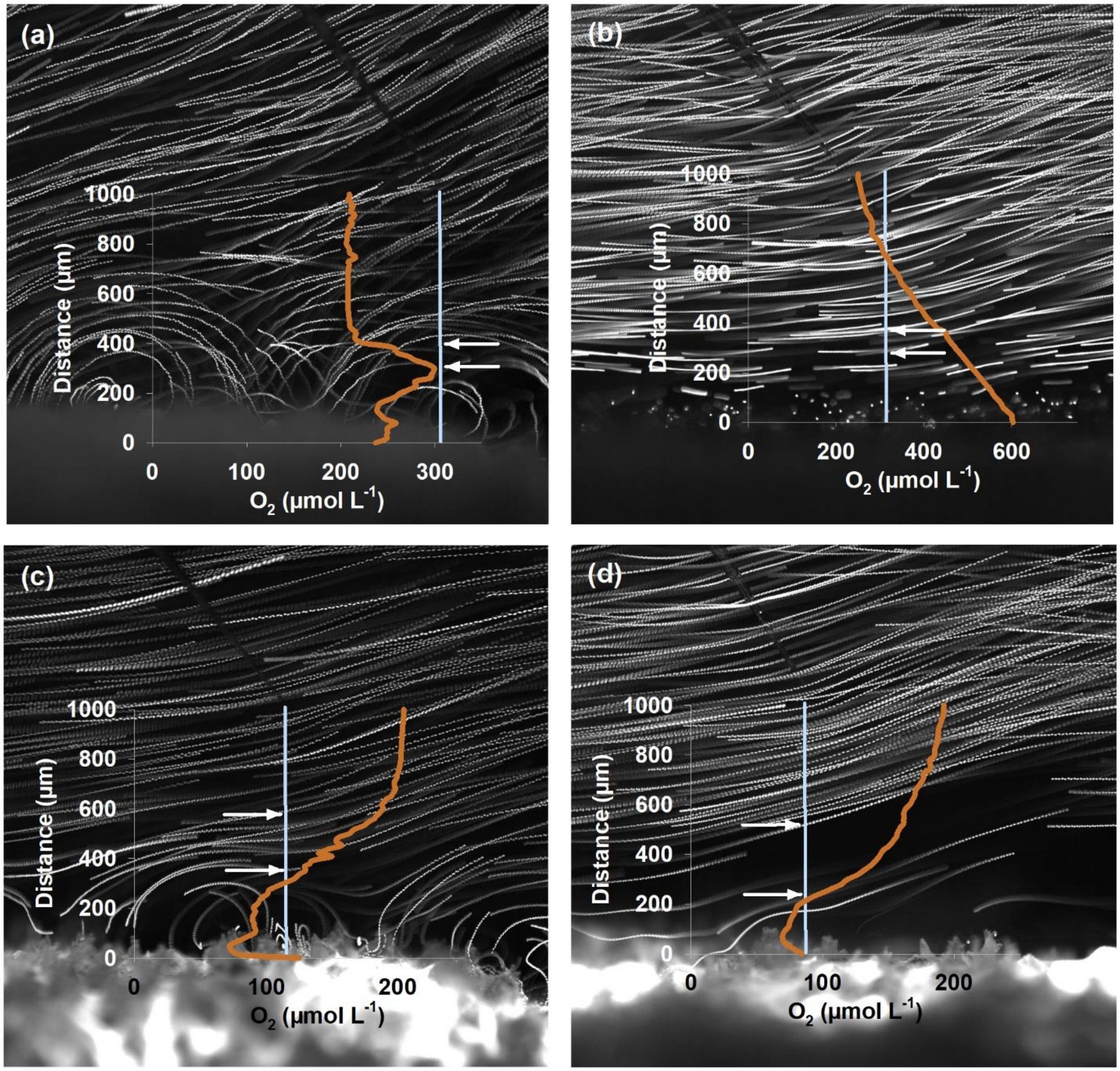

Fig. S2. PIV image and Oxygen profiles of the coral *Porites lutea* under active (**a**, **c**) and arrested (**b**, **d**) cilia activity as well as under Light (**a**, **b**) and Dark (**c**, **d**) conditions. Pathlines show particle trajectories. Orange lines indicate oxygen concentration along the light blue line measured with a microsensor (also seen in the images). Flow speed of water in this case was 300 µm s^{-1} measured at 2 mm from the coral surface (see text for more details). Arrows indicate the part of the DBL that was considered for oxygen flux calculations.

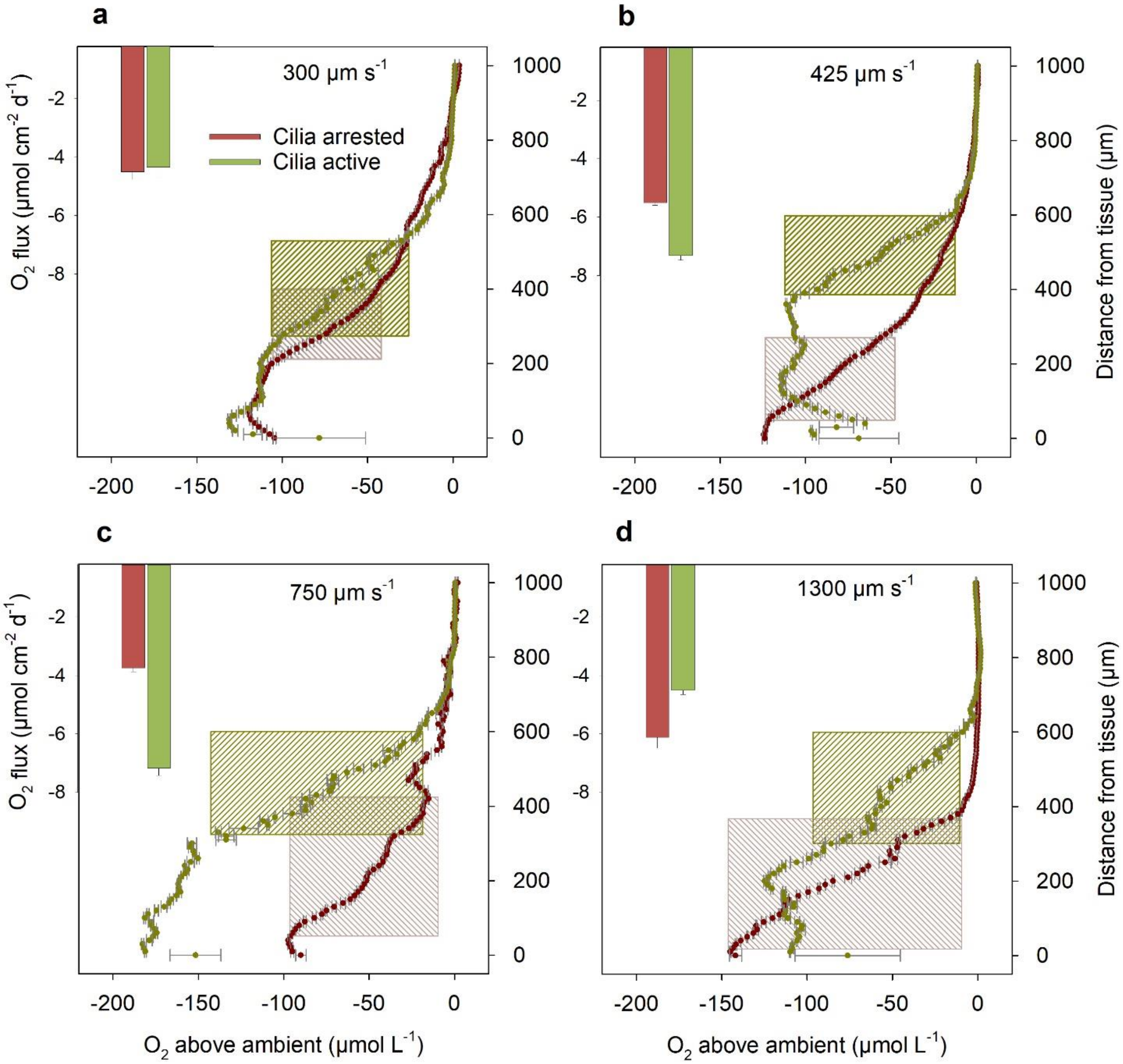

Fig. S3. Oxygen concentrations along a perpendicular 1000 µm transect from the tissue of *Porites lutea* to the water column in the dark, at flow speeds of (**a**) 300, (**b**) 425, (**c**) 750 and (**d**) 1300 µm s^{-1}, and for arrested and active cilia conditions. Inserted bar plots show the flux of oxygen in the DBL as calculated from oxygen gradients, either in the upper DBL (active cilia profile, green hatched background) or across a linear part of the DBL (arrested cilia profile, red hatched background). Error bars represent ± standard error of the linear regression.

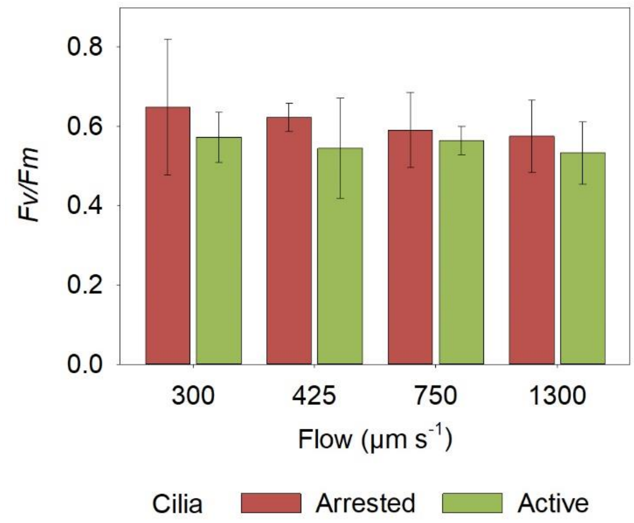

Fig. S4. Maximum quantum yield (MQY) of photosystem II (*Fv/Fm*) of zooxanthellae within *Porites lutea* in relation to the flow speed of the water (µm s^{-1}) and cilia activity (active and arrested) after 15 min of dark adaptation (0 µmol quanta m^{-2} s^{-1}). Error bars represent ±s.d.

Table S1. ANOVA results on the maximum quantum yield of photosystem II (*Fv/Fm*) of zooxanthellae within *Porites lutea* exposed to different treatment conditions regarding flow speed (µm s^{-1}), cilia activity (active and arrested) and light regime (dark, light).

Source of variation	Sum of squares	d.f.	Mean square	*F*	*P*
Light	19160	1	19160	2.627	0.115
Flow	7528	3	2509	0.344	0.794
Cilia	6604	1	6604	0.905	0.349
Light x Flow	3273	3	1091	0.150	0.929
Light x Cilia	12708	1	12708	1.742	0.196
Flow x Cilia	5336	3	1779	0.244	0.865
Light x Flow x Cilia	8853	3	2951	0.405	0.751
